# Trust and distrust: Identifying recruitment targets for ethnic minority blood donors

**DOI:** 10.1111/tme.12867

**Published:** 2022-05-02

**Authors:** Eamonn Ferguson, Erin Dawe‐Lane, Zaynah Khan, Claire Reynolds, Katy Davison, Dawn Edge, Susan R. Brailsford

**Affiliations:** ^1^ School of Psychology University of Nottingham Nottingham UK; ^2^ Leciestershire PartnershipTrust Mill Lodge, The Rise, Narborough Leicester UK; ^3^ NHS Blood and Transplant/UK Health Security Agency Epidemiology Unit London UK; ^4^ Division of Psychology and Mental Health University of Manchester Manchester UK

**Keywords:** blood donation, ethnicity, recruitment, trust

## Abstract

**Background:**

We explore the role of trust, distrust, and the prevailing socio‐political context to better understand why people from ethnic minority communities are less likely to be blood donors compared to people from White communities. Recruiting more ethnic minority donors will enhance representativeness, reduce inequality, and help meet the clinical need to increase the proportion of blood with Ro Kell antigen to treat Sickle Cell Disease (SCD).

**Study design and methods:**

A 2 (donor‐status: current donor; non‐donors) by 4 (ethnicity: People from Asian, Black, Mixed and White ethnic backgrounds) quasi‐experiment (*N* = 981) was conducted to examine perceptions of trust/distrust and their influence on willingness to donate blood, within the socio‐political context of the Windrush scandal and Brexit.

**Results:**

We identified five domains of trust (‘National Health Service [NHS] and staff,’ ‘NHS Blood and Transplant,’ ‘outgroups,’ ‘individuals’ and ‘politics’), and a single domain of conditional distrust domain. Trust across all the domains was lower, and ‘conditional distrust’ higher for ethnic minorities. Trust in ‘individuals’ and ‘NHSBT’ predicted willingness to donate in non‐donors from ethnic minorities and White non‐donors, respectively. Concerns about the Windrush scandal were related to lower political trust. Viewing Brexit as ‘positive for the UK’ was related to lower trust across domains and reduced willingness to donate in White non‐donors through its influence on reduced trust in NHSBT.

**Conclusion:**

Distinct domains of trust and distrust are identified, and targeting ‘trust in others’ through conditional cooperation is recommended as a strategy to increase donor numbers from ethnic minority communities.

## 1 INTRODUCTION

People from ethnic minorities are less likely to donate blood.^1^, ^2^, ^3^, ^4^ In England, for example, of those registered to donate blood in 2019–2020, blood donors from Black ethnic backgrounds made up 1.2% of all blood donations, and donors from Asian or Mixed ethnic backgrounds 2.1%. Greater diversity within blood donors can result in psychological (e.g., increased well‐being)^5^, ^6^ and clinical (e.g., improved treatment of sickle cell disease [SCD]) benefits.^1^, ^2^, ^3^, ^7^ For example, better outcomes for SCD are observed with donor‐recipient matching on Ro Kell antigens, which are more common in Black (52%) than White (2%)^1^, ^2^, ^3^ people. However, while demand for Ro antigen blood increases by 10%–15% each year, only 2% of blood donors in England have Ro antigens.^7^ Thus, a better understanding of why people from minority communities are less likely to donate blood will inform recruitment strategies that will help realise these potential benefits.^4^, ^8^, ^9^ To address this issue, this article focuses on the one key dimension known to influence interactions with healthcare in minority communities: *trust*.^4^, ^5^, ^6^

## 2 TRUST, ETHNICITY, HEALTHCARE AND BLOOD DONATION

While many barriers and motivators for donating blood are similar between minority and non‐minority donors and non‐donors,^10^, ^11^, ^12^, ^13^, ^14^ lower levels of trust in healthcare and donation services could partly explain the lower donation rates in ethnic minority communities.^4^, ^8^, ^9^, ^10^, ^11^, ^12^, ^13^, ^14^, ^15^, ^16^, ^17^, ^18^, ^19^, ^20^, ^21^ A lack of trust in medicine is also a demotivating factor for engaging with healthcare generally,^18^, ^19^ specifically for people from ethnic minority communities.^4^, ^15^, ^16^, ^20^, ^21^ Thus, a broader understanding of the role of trust in the context of blood donation should help to uncover new insights and inform recruitment strategies.^18^, ^22^

## 3 DOMAINS OF TRUST

Trust operates across many different domains in life.^23^ For example, people express varying degrees of trust in strangers (*individuals*),^24^, ^25^, ^26^ diverse communities, nationalities, and faiths (*outgroups*),^27^, ^28^ physicians,^18^, ^29^ and organisations of various types, including healthcare providers and the apparatus‐of‐states (e.g., police, judiciary, Government).^28^ These domains are all potentially important when individuals are considering donating blood. For example, blood donation is a public good, where a few donate blood to benefit all.^30^ A significant predictor of public good giving is trust in the generosity of *individuals* and members of *other groups*.^31^ Furthermore, historical betrayals of ethnic minority groups (e.g., Tuskegee, Windrush) reduce trust in the state (e.g., Government, law‐enforcement),^14^, ^32^, ^33^ which may undermine donation decisions, especially if the state and healthcare systems are perceived as linked.^14^ However, at present, the existing research on trust and blood donation has focused on a narrow set of domains, specifically trust in *healthcare* or *physicians*.^4^, ^8^, ^9^, ^10^, ^15^, ^16^, ^17^ To fully appreciate how trust impacts decisions about blood donation, we need to understand how trust (including trust outside the domain of healthcare) varies by ethnicity and donor status.

## 4 TRUST AND DISTRUST

It is essential to recognise that trust and distrust are separate constructs. While both function to reduce social complexity,^23^, ^34^, ^35^ trust creates positive expectations with desirable acts perceived with certainty.^25^, ^26^, ^36^, ^37^, ^38^ In contrast, distrust is not just a lack of trust but is linked to feelings that others are active harmful agents who cannot be relied on, leading to distrust, suspicion and alienation.^23^, ^35^

## 5 DONOR DECISION MAKING: WILLINGNESS TO INTENTIONS

Blood donors progress through a career from a non‐donor to a new/novice donor (one to four donations) to an experienced donor (five or more donations).^36^ Therefore, questions concerning decisions to donate blood need to be commensurate with the stage of the donor career being studied.^37^ For people who are inexperienced in a particular domain (e.g., blood donation), decisions are based on behavioural willingness (i.e., an individual's openness to behavioural opportunities and willingness to consider a behaviour); however, as the person becomes more experienced, decisions based on intentions become more important.^38^ As a primary focus of this article is to explore the predictive power of trust in non‐donors, behavioural willingness is assessed as the most appropriate decision‐making index.

## 6 SOCIO‐POLITICAL CONTEXT

Perceptions of trust and distrust are influenced by the contemporary cultural and political landscapes.^3^ However, previous work on trust and blood donation has not considered the influence of the broader socio‐political context. To account for the political context at the time of the study, we examined how perceptions of Brexit and the Windrush scandal influence trust in donors and non‐donors.

Brexit concerns the UK's exit from the European Union (EU) following a national referendum on the 23^rd^ of June 2016. This issue has dominated the political landscape in the UK since, leading to divided public opinion.^39^ The Windrush Scandal emerged in 2017, when hundreds of Black Commonwealth citizens, who came to the UK between 1948 and 1973 on their parent's passport, were erroneously classed as ‘illegal’ immigrants because the relevant documentation was lost. They were denied legal rights, detained, and deported.^34^

We test the conjecture that reduced trust in the political establishment is linked to perceiving leaving the EU as ‘a positive step for the UK.’^40^, ^41^ We explore if this generalises to concerns about the Windrush scandal and the broader domains of trust. Finally, we explore if the reduced level of trust reported by ethnic minorities is, in part, accounted for by their beliefs about Brexit and the Windrush Scandal.

## 7 AIMS OF THIS PAPER

This article explores how domains of trust (from individual to political) vary by ethnicity and donor status and whether they predict willingness to donate in non‐donors. Furthermore, we explore how trust and willingness to donate are associated with perceptions of Brexit and the Windrush Scandal.

## 8 METHODS

### 8.1 Design and sample frame

A 2 (*Donor status*: current vs. non‐donor) by 4 *(Ethnicity*: People from Asian, Black, Mixed and White ethnic backgrounds), quasi‐experiment was conducted. The donor sample was recruited from the UK National Health Service Blood and Transplant (NHSBT) database; a sample of 3500 people from ethnic minorities, and 2500 White people, who had donated in the last 2 years, were *randomly* selected. Non‐donors were primarily recruited through a market research company (Code 3: www.code3research.co.uk). A random sample of 4, 300 people from ethnic minorities and 4300 White people were selected (Supplementary File S1 for details, justification of sample sizes, and power calculations). Initial survey invitations were sent on the 14^th^ of June 2019, with a reminder 4 weeks later (12 July 2019). An additional reminder was sent to the ethnic minority sample on the 2^nd^ of August 2019. The study was designed to explore a wider set of variables (Supplementary File S2), but this paper focuses on trust. There was no payment for participating in the surveys. However, five participants from the Code 3 sample were selected at random to receive a £25 gift voucher.

#### 8.1.1 Current donor status

Current donors were defined as those who had given blood within the last 2 years. All donors recruited via the NHSBT database were selected to have donated in the last 2 years. However, all participants were asked if they had donated: (1) Less than a month ago, (2) 2–12 months ago, (3) 12 months to 2 years ago, (4) Longer than 2 years ago, (5) Cannot remember. Current donors from Code 3 were identified as those who reported one of: (1) Less than a month ago, (2) 2–12 months ago, (3) 12 months to 2 years ago. These participants were added to the current donors derived from the NHSBT sample.

#### 8.1.2 Coding ethnicity

Participants were sampled based on the ethnicity data recorded by NHSBT and Code‐3 (Supplementary File S1). Participants were also asked to self‐describe their ethnicity. These self‐descriptions were coded using the UK Office of National Statistics (ONS) criteria (Supplementary File Text S3, Supplementary Table S1). While there was a wide range of descriptions (Supplementary File S3), we coded these into the higher‐order ONS groups in terms of people from: (1) an *Asian* background (Indian, Pakistani, Bangladeshi, Chinese, any‐other‐Asian), (2) a *Black and Caribbean* background (African, Caribbean, any other Black/African/Caribbean), (3) a *Mixed Ethnic* background (White‐and‐Black‐Caribbean, White‐and‐Black‐African, White‐and‐Asian, Black‐and‐White, Arab, any‐other‐mixed) and (4) a *White* background (English/Welsh/Scottish/Northern Irish/British/Irish/other White). The White sample did not include any White minorities defined as Gypsy, Roma or Irish traveller groups (see Supplementary Files S3).

### 8.2 Measures

#### 8.2.1 Assessment of trust and distrust

Questions were derived from existing measures of trust to represent seven domains of trust in: (1) the UK National Health Service (NHS), (2) physicians, (3) National Health Service Blood and Transplant (NHSBT), (4) the equality of healthcare provision, (5) the apparatus of the state (police, courts, government), (6) outgroups and (7) individuals^24^, ^25^, ^26^, ^27^, ^29^, ^42^, ^43^, ^44^, ^45^, ^46^, ^47^, ^48^ (Supplementary File S4 details the items and supporting references). Each item was answered on a 5‐point scale, where higher scores equate to greater trust, except for trust in individuals,^25^ which was responded to on a 4‐point scale.

#### 8.2.2 Willingness to donate

Participants were asked, ‘Would you consider donating blood in the future?’ yes (1) or no (0).

#### 8.2.3 Socio‐politicalcontext

In terms of perceptions of Brexit, participants were asked: “Do you think Brexit is a positive or negative step for the future of the UK?” (positive [1] or negative [0]; 23.7% thought that Brexit was a positive step).

In terms of perceptions of the Windrush Scandal, participants were asked to what extent: “The Windrush Scandal shows that the authorities still have a negative view about ethnic minorities in the United Kingdom”? This was responded to with ‘not sure what this is,’ 1 = ‘strongly disagree’ to 5 = ‘strongly agree.’ Seventy‐four people (55% White people, 25% people from an Asian background, 17% people from a mixed ethnic background and 3% people from a Black and Caribbean background) stated that they were not sure what the Windrush Scandal was.

### 8.3 Statistical analyses

#### 8.3.1 Latent variable and path modelling

M*Plus* 8.4^49^ was used to specify factor analytic models to explore the dimension of trust and run path models. In all analyses, a diagonally weighted least squares with means and variance adjustment (WLSMV) extraction algorithm was used to account for the ordinal nature of these data. Fit statistics were used to assess the best fitting model, with the best model having a TLI and CFI >0.95 and RMSR of <0.05.^50^

#### 8.3.2 Exploratory factor analysis

While the items used to cover the domains of trust are derived mainly from existing measures, they have never been combined or applied in these samples or contexts. Under such circumstances, an exploratory approach has been recommended.^51^ As such, exploratory factor analysis was conducted on the trust items with Geomin rotation (Table 2), with an item classed as loading on a factor if it loaded 0.40 or greater.

#### 8.3.3 Exploratory path models

Path models were specified to examine if perceptions of Brexit and the Windrush Scandal indirectly linked ethnicity, age and sex to perceptions of trust, with perceptions of trust acting as proximal predictors of willingness to donate blood.

## 9 RESULTS

### 9.1 Sample characteristics

The final sample consisted of 981 participants (Table 1, Supplementary File S3).

**TABLE 1:** Sample characteristics

		All			
		*n* or mean	Non‐donors	Donors	Non‐donors versus donors
NHSBT donors	All ethnic minorities (excluding White minorities	376		376	
	White people	343		343	
Code 3 (market research)	All ethnic minorities (excluding White minorities	132	103	21	
	White people	122	111	19	
Community group	People from an Asian background	8	6	2	
Donor status	Current donors	761			
	Non‐donors	220			
Ethnicity					
	Asian	182	38 (17.3%)	144 (19.4%)	χ^2^_(3)_ = 24.43, *p* = 0.000. There were fewer donors from Black communities than expected. There were fewer non‐donors from Asian communities than expected
	Black	141	53 (24.1%)	88 (11.9%)	
	Mixed	182	27 (12.3%)	155 (20.9%)	
	White	456	102 (46.4%)	354 (48.8%)	
	Missing data	20			
Sex	Male	339	42 (19.3%)	297(39.4%)	χ^2^_(1)_ = 30.15, *p* = 0.000. There were more male donors than expected and fewer female non‐donors than expected
	Female Missing data	633 9	176 (89.7%) 2	457 (60.6%) 7	
Age		*M* = 44.65 (SD = 14.57) range 18–89	*M* = 46.05 (SD = 14.15)	*M* = 44.23 (SD = 14.67)	t (963) = 1.63, *p* = 0.193

*Note*: Current donors = donated within the last 2 years. Asian = People from Asian ethnic backgrounds, Black = People from Black and Caribbean backgrounds, Mixed = People from mixed ethnic backgrounds, White = People from White backgrounds (excluding White minorities).

### 9.2 The structure and dimensionality of trust

Results from the exploratory factor analysis are shown in Table 2. The amount of missing data was small (0.1%–0%) and missing completely random (Little's MCAR test: = (χ^2^_[480]_ = 519.53 *p* = 0.103). As such, missing data were treated using Full Information Maximum Likelihood (FIML). A six‐factor model best fit these data (TLI = 0.934, CFI = 0.967, RMSR = 0.038: Table 2), which was a better fit than a five‐factor model (χ^2^_[114]_ = 1367.05; *p* = 0.000), which was in turn a better fit than a 4‐factor model (χ^2^_[131]_ = 1961.38; *p* = 0.000). However, this six‐factor model did not conform to the primary scales, with combined and new factors observed, justifying the exploratory approach.

**TABLE 2:** Exploratory factor analysis of trust in donors and non‐donors

	Trust NHS and staff	Trust NHSBT	Trust individuals	Conditional distrust	Trust politics	Trust outgroup
I completely trust the National Health Services' (NHS) judgements about my medical care	**0.509** *	0.256*	0.031	−0.144*	0.101*	−0.120*
Patients receive high‐quality medical care from the National Health Service (NHS)	**0.543** *	0.185*	0.102*	−0.139*	0.035	−0.118*
I trust my GPs judgements about my medical care	**0.931** *	−0.058	−0.044*	0.073*	−0.011	0.085*
My GP would always tell me the truth about my health even if there was bad news	**0.795** *	−0.007	−0.035	0.019	−0.082*	0.143*
I feel respected by the National Health Service (NHS)	**0.633** *	0.154*	0.019	−0.055	0.008	−0.014
I trust the blood and transplant service to provide blood for all patients who need it.	0.023	**0.876** *	0.010	−0.002	−0.015	−0.054*
I trust the blood and transplant service to take care of blood donors	0.020	**0.967** *	−0.006	0.043*	−0.025	0.035*
I trust the blood and transplant service to screen blood to ensure it is safe.	−0.021	**0.945** *	−0.030	0.009	0.017	0.006
I trust the blood and transplant service to treat people from my ethnic group fairly	0.027	**0.840** *	−0.037	−0.115*	0.006	0.011
The National Health Service (NHS) experiments on patients without them knowing	−0.053	−0.177*	−0.049	**0.467** *	0.119*	−0.007
Rich patients receive better care in hospitals than poor patients	0.014	−0.149*	−0.088*	**0.562** *	−0.025	0.133*
People of my ethnic group cannot trust doctors and healthcare workers	−0.033	−0.173*	−0.089*	**0.656** *	0.063*	0.073
To what extent do you trust people from the police	0.009	−0.021	−0.042*	**−0.677** *	0.430*	0.140*
To what extent do you trust people from the courts	0.003	−0.040	−0.053*	**−0.665** *	0.534*	0.128*
To what extent do you trust people you meet for the first time	−0.036	−0.014	**0.852** *	−0.045	−0.010	0.082*
To what extent do you trust a stranger	0.028	−0.046	**0.845** *	0.061	0.012	−0.021
In general, one can trust people	0.077	0.141*	**0.448** *	−0.011	0.111*	0.079*
When dealing with strangers, it is better to be careful before you trust them:	0.024	0.074	**−0.626** *	−0.001	0.039	0.072
To what extent do you trust people of another religion	−0.005	0.024	0.468*	−0.092	0.003	**0.885** *
To what extent do you trust people of another nationality	0.013	0.027	0.464*	−0.028	0.014	**0.836** *
To what extent do you trust people from the government	−0.025	0.045	0.046*	−0.034	**0.893** *	−0.032
To what extent do you trust people from political parties	0.033	0.013	0.174*	0.074*	**0.812** *	−0.047*

	Latent correlations					
Trust in NHS & staff	1					
Trust NHSBT	0.626*	1				
Trust individuals	0.195	0.162*	1			
Conditional distrust	−0.484*	−0.491*	−0.231*	1		
Trust politics	0.215*	0.094*	0.193*	−0.168*	1	
Trust outgroup	0.107*	0.202*	−0.036	−0.163*	0.078	1

* *p* < .05.

The resultant factors were summed to create scales. As these scales are based on different numbers of items and some on a 5‐point and some on a 4‐point response format, scores were standardised to vary between 0 (no trust at all or complete lack of distrust) and 1 (complete trust or distrust) (Supplementary File 5).

The first factor focuses on trust in ‘NHS and Staff,’ measuring honesty and whether the NHS provides high‐quality care. The second factor, ‘Trust in NHSBT,’ reflects trust in the blood service to provide for patients, take care of blood donors and recipients, and ensure safety. The third factor, ‘Conditional Distrust,’ represented a belief that the NHS experiments on patients without their knowledge, that wealthy patients receive better care than poor patients, and that people from their ethnic community cannot trust NHS staff. This is combined with a general lack of trust in the police and judiciary. We term this ‘distrust’ as it reflects perceptions that others will actively harm the patient or person based on their ethnicity and social status (wealth) and, therefore, cannot be relied on.^21^, ^36^ The fourth factor, ‘Trust in Individuals,’ focuses on trust in strangers and encounters people have in their everyday lives. The fifth factor, ‘Trust in Outgroups,’ focuses on trust in people from other faiths and nationalities. The sixth factor, ‘Trust in Politics,’ reflects levels of trust in the Government and political parties.

### 9.3 Levels of perceived trust and distrust

The highest levels of trust were observed for ‘NHSBT’ (Mean = 0.85, SEM = 0.005; Mode = 1.0; *N* = 975), followed by the ‘NHS and staff’ (Mean = 0.72, SEM = 0.005; Mode = 0.75; *N* = 972), ‘Outgroups’ (Mean = 0.69, SEM = 0.005; Mode = 1.0; *N* = 977), ‘Individuals’ (Mean = 0.45, SEM = 0.006; Mode = 0.50; *N* = 966) and lowest in ‘Politics’ (Mean = 0.34, SEM = 0.008; Mode = 0.25; *N* = 974). Conditional distrust was also found to be relatively high (Mean = 0.29, SEM = 0.006; Mode = 0.25; *N* = 968).

**FIGURE 1:**
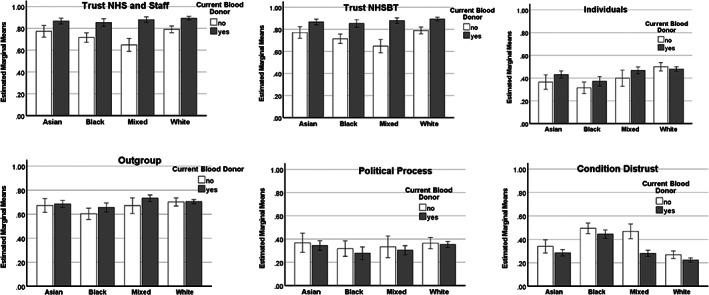
Dimensions of trust by donor status and ethnicity (error bars = 95% CIs)

Means scores for each standardised dimension of trust, split by ethnicity and donor status, are shown in Figure 1A–F (Supplementary File 6 for Tables).

### 9.4 Predictors of trust and distrust

Table 3 (Supplementary File S7, for sensitivity analysis) shows the results of a series of OLS regressions detailing the effects of sex, age, donor status, ethnicity and the interaction between donor status and ethnicity on the different domains of trust.

**TABLE 3:** Predictors of trust and distrust

	Trust								
	NHS & staff			NHSBT			Individuals		
	B	*p*=	95% CI	B	*p*=	95% CI	B	*p*=	95% CI
Age	−0.0008 (0.0005)	0.085	−0.0017, 0.0001	**−0.0009 (0.0004)**	**0.027**	**−0.002, −0.0001**	**0.003 (0.0005)**	**0.000**	**0.002, 0.004**
Sex	**0.043 (0.012)**	**0.000**	**0.019, 0.067**	0.009 (0.010)	0.442	−0.012, 0.030	**0.048 (0.014)**	**0.000**	**0.021, 0.075**
Current donor	**0.043 (0.021)**	**0.039**	**0.002, 0.084**	**0.011 (0.019)**	**0.000**	**0.061, 0.138**	−0.024 (0.022)	0.257	−0.067, 0.018
Ethnicity									
Asian	−0.071 (0.041)	0.088	−0.152, 0.010	−0.069 (0.035)	0.052	−0.139, 0.0005	**−0.100 (0.042)**	**0.017**	**−0.183, −0.018**
Black	**−0.078 (0.037)**	**0.034**	**−0.150, −0.006**	**−0.098 (0.039)**	**0.011**	**−0.174, −0.022**	**−0.156 (0.035)**	**0.000**	**−0.225, −0.088**
Mixed	**−0.108 (0.038)**	**0.004**	**−0.182, −0.034**	**−0.146 (0.043)**	**0.001**	**−0.231, −0.061**	−0.068 (0.040)	0.093	−0.149, 0.011
Interaction									
Donor*Asian	0.013 (0.043)	0.773	−0.073, 0.099	0.022 (0.038)	0.551	−0.052, 0.098	0.074 (0.075)	0.099	−0.014, 0.163
Donor*Black	0.001 (0.042)	0.979	−0.081, 0.083	0.051 (0.042)	0.222	−0.031, 0.135	**0.089 (0.089)**	**0.023**	**0.012, 0.167**
Donor*Mixed	**0.089 (0.040)**	**0.042**	**0.003, 0.160**	**0.109 (0.046)**	**0.017**	**0.019, 0.199**	**0.105 (0.044)**	**0.017**	**0.019, 0.191**
Brexit	**−0.034 (0.015)**	**0.008**	**−0.068, −0.009**	**−0.035 (0.014)**	**−0.012**	**−0.063, −0.008**	**−0.0423 (0.015)**	**0.004**	**−0.072, −0.138**
Windrush	−0.002 (0.007)	0.807	−0.016, 0.012	−0.004 (0.006)	0.531	−0.016, 0.08	−0.005 (0.007)	0.479	−0.19, 0.009
Constant	**0.760 (0.040)**	**0.000**	**0.681, 0.839**	**0.870 (0.038)**	**0.000**	**0.795, 0.944**	**0.357 (0.041)**	**0.000**	**0.276, 0.439**
R^2^	0.08			0.16			0.24		
*N*	854			857			850		

	Trust						Distrust		
	Outgroup			Political process			Conditional distrust		
	B	*p*=	95% CI	B	*p*=	95% CI	B	*p*=	95% CI
Age	0.005 (0.0004)	0.307	0.0004, 0.001	0.0006 (0.0006)	0.368	−0.0007,0.002	**−0.0008 (0.0004)**	**0.049**	**−0.002, −0.000003**
Sex	0.0007 (0.012)	0.951	−0.023, 0.025	−0.009 (0.018)	0.627	−0.044, 0.027	−0.002 (0.012)	0.881	−0.025, 0.022
Current donor	0.013 (0.020)	0.510	−0.026, 0.052	−0.004 (0.028)	0.892	−0.058, 0.051	**−0.053 (0.018)**	**0.004**	**−0.089, −0.017**
BAME status									
Asian	−0.064 (0.039)	0.089	−0.139, 0.010	0.042 (0.062)	0.490	−0.078, 0.163	0.050 (0.038)	0.200	−0.026, 0.124
Black	**−0.120 (0.036)**	**0.001**	**−0.190, −0.049**	0.003 (0.048)	0.944	−0.091, 0.098	**0.180 (0.038)**	**0.000**	**0.104, 0.256**
Mixed	−0.033 (0.035)	0.343	−0.103, 0.036	0.027 (0.052)	0.606	−0.075, 0.128	**0.159 (0.042)**	**0.000**	**0.076, 0.242**
Interaction									
Donor*Asian	0.020 (0.041)	0.626	−0.061, 0.101	−0.024 (0.064)	0.711	−0.151, 0.103	0.005 (0.040)	0.711	−0.074, 0.085
Donor*Black	0.046 (0.042)	0.275	−0.037, 0.129	−0.0003 (0.057)	0.995	−0.109, 0.109	−0.016 (0.044)	0.624	−0.103, 0.070
Donor*Mixed	0.050 (0.038)	0.186	−0.024, 0.125	−0.043 (0.056)	0.436	−0.151, 0.066	**−0.124 (0.044)**	**0.003**	**−0.211, −0.037**
Brexit	−0.027 (0.014)	0.052	−0.054, 0.0002	−0.0004 (0.019)	0.835	−0.045, 0.036	**0.055 (0.013)**	**0.023**	**0.029, 0.080**
Windrush	0.006 (0.006)	0.311	−0.006, 0.019	**−0.053 (0.010)**	**0.000**	**−0.073, −0.034**	**0.037 (0.006)**	**0.000**	**0.025, 0.049**
Constant	**0.671 (0.038)**	**0.000**	**0.594, 0.745**	**0.513 (0.057)**	**0.000**	**0.402, 0624**	**0.170 (0.035)**	**0.000**	**0.101, 0.240**
R^2^	0.06			0.053			0.16		
*N*	860			858			853		

*Note*: Sex (0 = female, 1 = male). Ethnicity: people from a White background are the comparison population. Current donor (0 = No, 1 = Yes), Brexit (0 = negative influence, 1 = positive influence). Windrush (“The Windrush Scandal shows that the authorities still have a negative view about ethnic minorities in the United Kingdom” 1 = ‘strongly disagree’ to 5 = ‘strongly agree.’) Asian = People from Asian ethnic backgrounds, Black = People from Black and Caribbean backgrounds, Mixed = People from mixed ethnic backgrounds, White = People from a White backgrounds (excluding White minorities).

Men are more trusting than women with regards to ‘NHS and Staff’ and ‘Individuals.’ Current donors are more trusting of the ‘NHS and Staff’ and ‘NHSBT’ and express lower ‘Conditional Distrust’ than non‐donors. Older participants were more trusting of ‘Individuals’ and had lower ‘Conditional Distrust’ and trust in NHSBT. Those who viewed Brexit as a ‘positive benefit for the UK’ were less trusting of the ‘NHS and Staff,’ ‘NHSBT,’ ‘Individuals’ and displayed higher ‘Conditional Distrust.’ Therefore, it could be suggested that those who saw the UK leaving the EU as a benefit were less trusting of UK systems that could be construed as supporting the campaign to remain in the EU.^43^, ^44^ Concerns about the Windrush scandal were associated with reduced trust in politics and greater conditional distrust (Supplementary File S8 for more detail on cultural context).

There are several significant effects of ethnicity. People from Asian ethnic backgrounds had less trust in ‘Individuals’ than White people. People from a Black ethnic background had less trust in ‘NHS and Staff,’ ‘NHSBT,’ ‘Individuals’ and ‘Outgroups,’ and expressed greater ‘Conditional Distrust’ than White people. Finally, compared to White people, people from mixed‐ethnic backgrounds had less trust in ‘NHS and Staff’ and ‘NHSBT’ and expressed greater ‘Conditional Distrust.’

The effects of donor status and ethnicity were qualified by a series of significant interactions for trust in ‘NHS and Staff,’ ‘NHSBT,’ ‘Individuals’ and ‘Conditional Distrust.’ These interactions were explored using *margins* in Stata 16 (Supplementary File Text S7 for the full margin table relating to Table 3 and for sensitivity analysis). These show that compared to non‐donors, donors from White or mixed‐ethnic backgrounds had greater trust in ‘NHS and Staff.’ Compared to non‐donors, donors from Asian, Black, mixed‐ethnic or White backgrounds had greater trust in ‘NHSBT.’ Compared to non‐donors, donors from mixed‐ethnic backgrounds had greater trust in ‘Individuals.’ Donors from White or mixed‐ethnic backgrounds have lower ‘Conditional Distrust’ than non‐donor.

### 9.5 Indirect effects of socio‐politicalfactors

We explored if the perception of Brexit and the Windrush scandal indirectly linked demographics (age, sex and ethnicity) to the domains of trust (Supplementary File 9 for model fit, and detailed results). In summary, perceptions that Brexit is ‘likely to be beneficial for the UK’ was the mechanism linking increased age to low trust in ‘NHS and Staff,’ ‘NHSBT,’ ‘individuals’ and ‘outgroups’ and greater ‘conditional distrust.’ Perceptions that the Windrush Scandal indicated that ‘the UK government holds negative views of people from ethnic minority backgrounds’ linked being a woman and/or being from an ethnic minority community to low trust in individuals, politics and greater conditional distrust.

### 9.6 Predicting donation willingness in non‐donors

Table 4 details two exploratory logistic regression models that examine predictors of willingness to donate in non‐donors. The first (columns 2 and 3) explores the effects of age, sex, ethnicity, and the interaction of ethnicity by trust. The second (columns 4 and 5) includes the effects of Brexit and the Windrush scandal. The results show that younger non‐donors were more willing to donate and that overall, trust in NHSBT predicted greater willingness to donate.

**TABLE 4:** Logistic regression for willingness to donate in non‐donors

	B (S.E.)	*p*=	B (S.E.)	*p*=
**Age**	**−0.104 (0.026)**	**0.000**	**−0.114 (0.030)**	**0.000**
Sex	0.812 (0.733)	0.268	0.859 (0.786)	0.275
Ethnicity	7.200 (4.483)	0.108	8.243 (5.069)	0.104
Trust NHS and staff	0.692 (3.025)	0.819	0.985 (3.120)	0.752
**Trust NHSBT**	**5.899 (2.788)**	**0.034**	**7.124 (3.348)**	**0.033**
Condition distrust	3.558 (2.818)	0.207	4.686 (3.213)	0.145
Trust individuals	−1.082 (2.488)	0.664	−0.320 (2.535)	0.900
Trust outgroup	4.379 (2.911)	0.133	3.695 (3.154)	0.241
Trust politics	1.556 (1.782)	0.383	1.122 (1.889)	0.552
Ethnicity* Trust NHS and staff	2.299 (3.681)	0.532	1.944 (3.751)	0.604
Ethnicity* Trust NHSBT	**−7.289 (3.650)**	**0.046**	**−8.407 (4.180)**	**0.044**
Ethnicity*Condition distrust	−2.678 (3.726)	0.472	−3.095 (4 0.100)	0.450
Ethnicity* Trust individuals	**6.763 (3.359)**	**0.044**	**6.654 (3.431)**	**0.052**
Ethnicity*Trust outgroup	−6.696 (3.715)	0.072	−6.313 (3.977)	0.112
Ethnicity*Trust politics	−2.824 (2.408)	0.241	−2.568 (2.496)	0.304
Brexit			−0.113 (0.667)	0.866
Windrush Scandal			−0.297 (0.323)	0.358
Constant	−2.070 (3.425)	0.546	−1.596 (4.318)	0.712
R^2^	0.362		0.395	
*n*	176		164	

*Note*: Sex (0 = female, 1 = male). Ethnicity: People from a White background are the comparison population. Brexit (0 = negative influence, 1 = positive influence). Windrush (“The Windrush Scandal shows that the authorities still have a negative view about ethnic minorities in the United Kingdom” 1 = ‘strongly disagree’ to 5 = ‘strongly agree’). White minorities are not represented.

There were two significant moderating effects of ethnicity on trust, one for NHSBT and one for trust in individuals. The margins for these interactions are in Tables S8 and S9 in Supplementary File S10. These show that greater ‘trust in individuals’ predicts willingness to donate for people from ethnic minority backgrounds (Table S8) and that trust in NHSBT predicts willingness to donate in people from White communities (Table S9).

**FIGURE 2:**
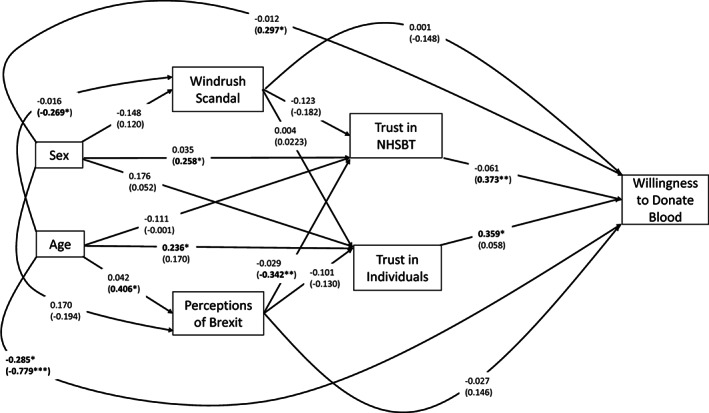
Path model to represent the downstream effects of age, sex, perceptions of Brexit and the Windrush Scandal on trust and willingness to donate blood in non‐donors. *p < 0.05, **p < 0.01, ***p < 0.001. Parameter estimates for people from all ethnic minority backgrounds are the upper coefficients not in parentheses. The parameter estimates for people from White backgrounds are the lower coefficients in parentheses (*n* for the ethnic minority people is 96, and for the White people, *n* is 71)

Finally, an overall path model to summarise the main predictor of willingness to donate in non‐donors was specified (Figure 2). This model included the two main trust dimensions (NHSBT and individuals) predicting willingness in non‐donors and the potential indirect effects of demography on trust via indirect paths such as perceptions of Brexit and the Windrush (See Supplementary File S11 for full details). This model shows that ‘trust in individuals’ predicts willingness to donate in ethnic minority people and ‘trust in NHSBT’ for people from White communities. There is also evidence of a potential indirect effect of Brexit on willingness to donate through its influence on “trust in NHSBT’ for White people, such that perceiving Brexit as a positive move for the UK’ was linked to lower “trust in NHSBT” and through this, reduced willingness to donate blood (β_standardised_ = −0.130, *p* = 0.083).

## 10 DISCUSSION

This article demonstrates why a broader conceptualisation of trust is, in part, important for understanding why people from ethnic minority communities are less likely to donate blood. We explored these findings and their implications below.

### 10.1 Trust, distrust and blood donation

The results show a clear differentiation between trust and distrust, with trust separating into five domains: (1) politics, (2) healthcare organisations and their staff (e.g., NHS), (3) blood services (e.g., NHSBT), (4) outgroups (e.g., peoples of other nationalities and religions), and (5) individuals (e.g., strangers). The results show that people do not differentiate healthcare organisations (NHS) and their staff. This may reflect the uniqueness of the UK health service with a single national organisation employing medical staff, and staff are seen as representatives of that organisation. In other countries, with private healthcare providers, the link between the healthcare organisation and staff may be less clear. However, trust in the blood service (NHSBT) was seen as separate from the NHS. Thus, while NHS and NHSBT are related organizationally, psychologically, they are considered distinct.

A separate conditional distrust^23^ factor emerged that linked the idea that healthcare providers may actively harm patients or treat them differentially based on their ethnicity and wealth, combined with low trust in the police or judiciary.^14^ This conditional distrust can lead to a culture of distrust, suspicion, and alienation,^23^, ^35^ and is important as it shows a clear link between distrust in healthcare and the apparatus of the states (e.g., police and judiciary). Together, this indicates that reducing distrust in the healthcare system is not as simple as targeting interventions on healthcare but involves a broader consideration of distrust in society. Thus, widespread societal interventions that target distrust are needed, and blood services should consider working with outside government agencies to bring about effective change.

People from ethnic minorities, regardless of their blood donor status, reported significantly less trust across the domains, especially people from Black and Caribbean backgrounds. Lower levels of trust expressed by ethnic minorities were not only focused on organisations but also on individuals.^17^, ^52^ It should be noted that while ethnic minorities had lower trust in ‘NHS and Staff’ and ‘NHSBT’ compared to White people, levels of trust were still extremely high. Nevertheless, this was not the case for trust in ‘individuals,’ which was lower for all participants and especially people from Black and Caribbean backgrounds.

Additionally, ‘Conditional Distrust’ was higher in people from Black and Caribbean communities. This may reflect the ‘hostile environment’ around migration and the implications of Brexit.^53^ Indeed, concerns about the Windrush scandal were associated with higher ‘conditional distrust.’ It is often reported that the distrust that may arise from historical betrayals and distrust in various institutions and the apparatus of the state (police and courts) are key features of conditional distrust.^17^, ^23^

### 10.2 Implications for donor recruitment from minority communities

Trust in ‘individuals’ predicted willingness to donate blood for non‐donors from all ethnic minorities, which has clear implications for interventions. Critical here is the idea of conditional cooperation.^54^ Conditional cooperation occurs when people are aware that other people are cooperating, which motivates them to cooperate.^54^ As such, conditional cooperation is a powerful phenomenon that could be harnessed to increase cooperative behaviour, such as blood donation.^55^ One way to achieve this is via social media status updates such as—‘I have just donated blood’ or a blood donation status icon on Facebook, WhatsApp or Instagram, which would inform people that the individual has just donated blood and thereby encourage others to consider donating blood. This approach is effective in increasing opt‐in organ donor registrations.^56^ Thus, conditional cooperation may be particularly effective at recruiting non‐donors as it is a strong social force when free‐riding is high, which is the case for blood donation.^57^

### 10.3 Caveats

We showed that ‘Trust in Individuals,’ not trust in healthcare, predicts willingness to donate in non‐donors from ethnic minority communities. However, we must acknowledge that we grouped ethnicity into broad categories, minimising any effect of heterogeneity and wider diversity. Furthermore, the sample sizes for the analyses supporting the moderation and mediation analyses are small, and as such underpowered.^58^ Thus, while this work offers a starting point, it needs to be refined to explore trust and concomitant interventions in different ethnic communities and replicated in larger samples and cross‐validated with other methods.

## CONFLICT OF INTEREST

None of the authors have declared any conflicts of interest.

## Supporting information


**Supplementary File S1**Sampling procedure, power calculations and expected response rates
**Supplementary File S2**: Wider structure of the survey
**Supplementary File S3**: ONS categorization
**Supplementary File S4**: Proposed domains of trust derived from existing measures
**Supplementary File S5**: Standardising trust scores
**Supplementary File S6**: Standardised trust scores as a function of ethnicity and donor status
**Supplementary File S7**: Analysis of margins and sensitivity analyses
**Supplementary File S8**: Analysis of the cultural context of the study: Brexit & Windrush Scandal
**Supplementary File S9**: Details of path models for the indirect effect of perceptions of the Brexit and the Windrush Scandal on the demographic‐trust link
**Supplementary File S10**: Examination of the interaction of ethnicity by trust to predict willingness to donate in non‐donors
**Supplementary File S11**: Detail of summary path models for the prediction of willingness to donate in non‐donors

## Data Availability

Data reported in this article is available from the first author on request.
