# Supplementary material for: Trust and distrust: Identifying recruitment targets for ethnic minority blood donors
Source: Transfus Med. 2022 May 2;32(4):276–87. doi: 10.1111/tme.12867 (PMC9542243; doi:10.1111/tme.12867)
Supplement: Supplementary file 1 — Supplementary File S1Sampling procedure, power calculations and expected response rates Supplementary File S2: Wider structure of the survey Supplementary File S3: ONS categorization Supplementary File S4: Proposed domains of trust derived from existing measures Supplementary File S5: Standardising trust scores Supplementary File S6: Standardised trust scores as a function of ethnicity and donor status Supplementary File S7: Analysis of margins and sensitivity analyses Supplementary File S8: Analysis of the cultural context of the study: Brexit & Windrush Scandal Supplementary File S9: Details of path models for the indirect effect of perceptions of the Brexit and the Windrush Scandal on the demographic‐trust link Supplementary File S10: Examination of the interaction of ethnicity by trust to predict willingness to donate in non‐donors Supplementary File S11: Detail of summary path models for the prediction of willingness to donate in non‐donors [file TME-32-276-s001.docx]

**Supplementary Files for “Trust and Distrust: Identifying Targets for Recruitment of Ethnic Minority Donors.”**

Table of Contents

[Supplementary Files S1: Sampling procedure, power calculations and expected response rates 2](#_Toc101522773)

[**Sampling Procedures** 2](#_Toc101522774)

[**Power and Expected Response Rate** 3](#_Toc101522775)

[Supplementary File S2: Wider Structure of the Survey 5](#_Toc101522776)

[Supplementary File S3: ONS Categorization 6](#_Toc101522777)

[Supplementary File S4: Proposed Domains of Trust Derived from Existing Measures 7](#_Toc101522778)

[Supplementary File S5: Standardizing Trust Scores 10](#_Toc101522779)

[Supplementary File S6: Standardized Trust Scores as a Function of Ethnicity and Donor Status 11](#_Toc101522780)

[Supplementary File S7: Anaysis of Margins and Sensitivity Analyses 13](#_Toc101522781)

[Supplementary File S8: Analysis of the Cultural Context of the Study: BREXIT & Windrush Scandal 19](#_Toc101522782)

[Supplementary File S9: Details of Path Models For the Indirect Effect of Perceptions of the Brexit and the Windrush Scandal on the demographic-trust link 20](#_Toc101522783)

[Supplementary File S10: Examination of the interaction of ethnicity by trust to predict willingness to donate in non-donors 26](#_Toc101522784)

[Supplementary File S11: Detail of Summary Path Models For the Prediction of Willingness to Donate in Non-Donors 27](#_Toc101522785)

# Supplementary Files S1: Sampling procedure, power calculations and expected response rates

This section details the sampling procedures used, the power calculations, and expected response rates.

## **Sampling Procedures**

**NHSBT:** ethnic minorities

A random sample of 3,500 ethnic minorities (including White minorities) and 2,500 donors from White backgrounds were recruited, as long as they had not opted out of NHSBT communications or had medical deferrals and had not been sampled in other NHSBT research/surveys in the last 6 months.

Participants were sampled if they: (1) were aged over 18, (2) had registered to donate blood and had donated in the last two years, as we are interested primarily in current active donors (this is how NHSBT defines a current active donor, those who have not donated for two years are archived as lapsed donors), (3) self-identified by their reported ethnic group as an ethnic minority (excluding White minorities) or people from a White background (including White minorities) based on self-reported PULSE codes (e..g., Caribbean, African, Any other Black/African/Caribbean background, MIXED White and Black Caribbean, White and Black African and English/Welsh/Scottish/Northern Irish/British, Irish, Gypsy or Irish Traveller) .and (4) had an e-mail address registered with NHSBT and not shared by 2 or more donors. Both male and female donors were sampled.

**Code 3:**

From the Code 3 database of 100,059 participants, a random sample of 4,300 ethnic minorities (excluding White minorities) (from 12,727) and 4,300 people from a White background (from 65,078) participants were sampled. Participants’ were sampled if they: (1) were aged over 18, (2) registered with Code 3 and had not withdrawn permission to be contacted, and (3) identified as ethnic minorities (excluding White minorities) (African or Caribbean, White/Black African and White/Black Caribbean, Indian and Pakistani and any other Asian background) or people from a White background (White British and White Irish and other White) group member.

**Community Group Booster Samples:**

We also recruited ethnic minority (excluding White minorities) people from several local community groups if they were: (1) aged over 18, (2) registered on the community groups email list who have given consent to be contacted by the community and had not withdrawn permission.

## **Power and Expected Response Rate**

Within the African American community in the USA, trust in hospitals is positively associated with becoming a blood donor with an Odds Ratio of 2.32: [James, Schreiber, Hillyer, Shaz. 2013]). This equates to a Cohen’s d of 0.46 and an *r* of 0.22, both of which are small effect sizes. In the general public, ‘fear of hospitals’ was associated with a 2-fold decrease in prior history of being a blood donor [[Boulware](https://onlinelibrary.wiley.com/action/doSearch?ContribAuthorStored=Boulware%2C+LE), Ratner, Ness, Cooer, Campbell-Lee, LaVeist, Powe, 2002], equating to a Cohen’s d of 0.38 and an *r* of 0.19, Thus, we base our power calculations on a small effect size (Cohen’s d = .50 and r = .10). All power calculations are set at 80% power, with an alpha of .05 and are two-tailed and conducted in ZumaStat and G*Power. To do our basic analysis comparing across the groups would require 393 per group. Regression models with seven predictors (facets of trust) would require a total N of 998.

Based on previous NHSBT/PHE (now UKHSA) surveys, we expected a 15% response rate from donors from ethnic minorities (excluding White minorities) and a 21% response rate from donors from people from White backgrounds. Thus, a random sample of donors from 3,500 ethnic minorities (excluding White minorities) and 2,500 people from White backgrounds was sampled from the NHSBT database. Code 3 staff indicated an expected 12% response rate from ethnic minorities (excluding White minorities) and people from White backgrounds. Similarly, we aimed to recruit 500 people from White backgrounds and ethnic minorities (excluding White minorities) as a sample of primarily non-donors. Thus a random sample of 4,300 ethnic minorities (excluding White minorities) (from 12,727) and 4,300 people from White backgrounds (from 65,078) were sampled.

**Reference**

[Boulware](https://onlinelibrary.wiley.com/action/doSearch?ContribAuthorStored=Boulware%2C+LE) LE, Ratner LE, Ness PM, Cooer LA, Campbell-Lee S, LaVeist TA. Powe. The contribution of sociodemographic, medical, and attitudinal factors to blood donation among the general public. *Transfusion* 2002; 42: 669-678

James AB, Schreiber GB, Hillyer CD, Shaz, BH. Blood donations motivators and barriers: a descriptive study of African American and white voters. *Transfus Apher Sci* 2013; 48: 87-93

# Supplementary File S2: Wider Structure of the Survey

The survey had the following sections.

| **Area** | **Focus** |
| --- | --- |
| **Demographics** | Age, sex, self-defined ethnicity, |
| **Previous Donor Status** | Have you ever donated blood? Yes No  If yes – was this in the UK or abroad? UK Abroad  If yes – when was the last time you donated? Less than a month ago, 2 to 12 months ago, 12months to 2years ago, Longer than 2 years ago, Can’t remember |
| **Family Donate Blood** | Do you know any people from the following groups who have donated blood? Your family, your friends, your work colleagues, your neighbours |
| **Willingness to Donate** | Would you consider donating blood in the future?  Yes No |
| **Awareness** | Awareness of the need for BAME blood and awareness of NHSBT campaigns |
| **Trust** | Questions derived to assess trust in (1) NHS and UK health care, (2) NHSBT, (3) other groups (nationalities, religions), (4) organisation (police, courts, government), and (5) strangers and (7) items measuring distrust based on ethnicity and social class (see main text for details) |
| **Altruism** | We asked participants to respond to 4 hypothetical dictator games (DGs) to assess altruism. For each DG, we asked participants to split £50 between themselves and (1) a relative, (2) a stranger, (3) an overseas charity, and (4) a local community event. |
| **Social Isolation** | We asked about social isolation within the participant’s local community and the UK in general. |
| **Racial Discrimination** | We asked about racial discrimination within the health service specifically and in general. |
| **Donor Health Check** | We asked participants to consider and evaluate three questions from the UK Donor Health Check (DHC). These questions focused on (1) having sex with someone from areas with a high prevalence of HIV/AIDS, (2) returning from an area where there is malaria, including many parts of Africa, Asia, and South America in the last four months they are asked not to donate and (3) if they were born or ever lived or stayed outside the UK for a continuous period of 6 months or more. |
| **Socio-Political Context** | We asked whether people perceived Brexit as a positive move for the UK and awareness of the Windrush Scandal and the extent to which participants perceive that it indicates that the UK Government has a negative view of ethnic minorities. |

# Supplementary File S3: ONS Categorization

Below are the ONC categorisation. The white sample does not include white minorities (e.g, Gypsy, Roma or Irish Traveller groups or specified as ‘White other’).

**Table S1:** *Coding of Self-Defined Ethnicity*

|  | n | Valid Percentage |
| --- | --- | --- |
|  |  |  |
| **People from Asian ethnic backgrounds** |  |  |
| Indian | 61 | 6.3% |
| Pakistani | 19 | 2.0% |
| Bangladeshi | 5 | 0.5% |
| Chinese | 1 | 0.1% |
| Any other Asian background, please describe | 96 | 10% |
|  |  |  |
| **People from Black and Caribbean backgrounds** |  |  |
| African | 35 | 3.6% |
| Caribbean | 32 | 3.3% |
| Any other Black/African/Caribbean background, please describe | 74 | 7.7% |
|  |  |  |
| **People from mixed ethnic backgrounds** |  |  |
| White and Black Caribbean | 39 | 4.1% |
| White and Black African | 10 | 1% |
| White and Asian | 37 | 3.9% |
| Any other Mixed/Multiple ethnic backgrounds, please describe | 69 | 7.2% |
| Black & White | 3 | 0.3% |
|  |  |  |
| **Other ethnic groups** |  |  |
| Arab | 8 | 0.8% |
| Any other ethnic group, please describe | 16 | 1.7% |
|  |  |  |
| **White People** |  |  |
| English/Welsh/Scottish/Northern Irish/British | 426 | 44.3% |
| Irish | 3 | 0.3% |
| Gypsy or Irish Traveller | 0 | 0% |
| Any other White background, please describe | 27 | 2.3% |
|  |  |  |
| Missing | 20 |  |

Any other White background included White/Anglo Saxon, White European, White Caucasian, White Jewish; Any other Mixed/Multiple ethnic backgrounds included ‘English, Caribbean, Indian’, ‘White British and African American’,’ White, Black Caribbean and Asian, ‘White and Guyanese (Indian-Caribbean)’, ‘white parent & mixed white/Caribbean parent’, ‘Anglo-Hispanic]; Any other Asian background included ‘Asian – Iranian, ‘Asian Japanese’, ‘British Indian – Punjabi’, ‘Burmese’, ‘Bengali’; Any other Black/African/Caribbean background included ‘Black British Caribbean’, ‘African American/Black’, ‘English & Jamaican’, ‘British Anglo Afro American’; Any other ethnic group included ‘Arab- mixed Black and White African, ‘Egyptian.’

# Supplementary File S4: Proposed Domains of Trust Derived from Existing Measures

Based on the scales developed and reported in the literature, the following items were selected to represent the seven theoretical broad domains of trust we aimed to assess

1. **Trust in the National Health Service (Trust in Healthcare System)**

The following statements were selected to assess trust in the healthcare system overall. These were scored on a 5 point scale (where 1 = strongly disagree, 7 strongly agree) and were taken from Shea et al., (2008) and Cunningham et al. (2007) with the item ‘I feel respected by the National Health Service (NHS)’ suggested by Professor Barbra Masser (an external advisor on the survey).

- Patients receive high-quality medical care from the National Health Service (NHS).
- I completely trust the National Health Services’ (NHS) judgments about my medical care.
- The National Health Service (NHS) experiments on patients without them knowing.
- I feel respected by the National Health Service (NHS)

Shea,A,J., Micco,E., Dean,T,L., McMurphy,S., Schwartz,S,J., & Armstrong,K. (2008) Development of a Revised Healthcare System Distrust Scale. *Journal of General Internal Medicine*, 23: 727–732. https://doi.org/10.1007/s11606-008-0575-3

Cunningham,O,C., Sohler,L,N., Korin,L., Gao,W., Anastos,K. (2007) HIV status, trust in healthcare providers, and distrust in the healthcare system among Bronx women. *Aids Care*, 226-234. https://doi.org/10.1080/09540120600774263

1. **Trust in Physicians**

The following statements were selected to assess trust in individual physicians. These were scored on a 5 point scale (where 1 = strongly disagree, 7 strongly agree), and were taken from Thom et al., (1999) and Safran et al, (1998)

- I trust my GPs judgments about my medical care
- My GP would always tell me the truth about my health even if there was bad news

Thom et al., (1999) Further validation and reliability testing of the trust in Physician scale. Medical Care, (37),5,510-517.

Thom, D. Ribisl, K. Stewart, A., Luke, D; (1999) Further validation and reliability testing of the trust in Physician scale. *Medical Care,* 37,510-517. https://doi.org/10.1097/00005650-199905000-00010

[Safran DG](https://www.ncbi.nlm.nih.gov/pubmed/?term=Safran%20DG%5BAuthor%5D&cauthor=true&cauthor_uid=9596063), [Kosinski M](https://www.ncbi.nlm.nih.gov/pubmed/?term=Kosinski%20M%5BAuthor%5D&cauthor=true&cauthor_uid=9596063), [Tarlov AR](https://www.ncbi.nlm.nih.gov/pubmed/?term=Tarlov%20AR%5BAuthor%5D&cauthor=true&cauthor_uid=9596063), [Rogers WH](https://www.ncbi.nlm.nih.gov/pubmed/?term=Rogers%20WH%5BAuthor%5D&cauthor=true&cauthor_uid=9596063), [Taira DH](https://www.ncbi.nlm.nih.gov/pubmed/?term=Taira%20DH%5BAuthor%5D&cauthor=true&cauthor_uid=9596063), [Lieberman N](https://www.ncbi.nlm.nih.gov/pubmed/?term=Lieberman%20N%5BAuthor%5D&cauthor=true&cauthor_uid=9596063), [Ware JE](https://www.ncbi.nlm.nih.gov/pubmed/?term=Ware%20JE%5BAuthor%5D&cauthor=true&cauthor_uid=9596063). (19988).The Primary care Assessment Survey: tests of data quality and measurement performance. *Medical Care*, 36:728-739.

1. **Trust in National Health Service Blood and Transplant (NHSBT) – the transfusion service**

The following statements were selected to assess trust in NHSBT. Participants read the following description – “NHS Blood and Transplant are a separate part of the National Health Service that recruit and screen blood donors to ensure their blood is safe, run blood donation sessions and provide blood to the National Health Service. Can you please indicate the extent to which you agree or disagree with each statement below about NHS Blood and Transplant?” These were scored on a 5 point scale (where 1 = strongly disagree, 7 strongly agree), and were developed by the research team.

- I trust the Blood and Transplant Service to provide blood for all patients who need it.
- I trust the Blood and Transplant Service to take care of blood donors.
- I trust the Blood and Transplant Service to screen blood to ensure it is safe.
- I trust the Blood and Transplant Service to treat people from my ethnic group fairly.

1. **Trust in the Equality of Healthcare Provision**

The following statements were selected to assess the patient's trust in the healthcare system. These were scores on a 5 point scale (where 1 = strongly disagree, 7 strongly agree), and were taken from Boulware et al., (2002) and Thompson et al.,(2004).

- Rich patients receive better care in hospitals than poor patients.
- People of my ethnic group cannot trust doctors and healthcare workers

Boulware, L, E., Ratner, L, E., Sosa, J, A., Cooper, L, A., LaVeist, T, A., Powe, N, R.(2002) Determinants of willingness to donate living related and cadaveric organs: identifying opportunities for intervention. *Transplantation*, 27;73(10):1683-91. <https://doi.org/10.1097/00007890-200205270-00029>

Thompson,S,H.,Valdimarsdottir,B,H.,Winkel,G.,Jandorf,L.,Redd,W.(2004) The Group-Based Medical mistrust scale; psychometric properties and association with breast cancer screening. *Preventive Medicine*, 38:209-218. https://doi.org/10.1016/j.ypmed.2003.09.041

1. **Trust in the Apparatus of the State**

The following statements were selected to assess trust in the apparatus of the State. Participants were asked – “Could you tell us for each group listed below the extent to which you trust people from each group?”. These were scored on a 5 point scale where 1 = Do not trust at all, 7 Trust completely), and were taken from Newton and Zmerli (2011)

- The police
- The courts
- The Government
- Political Parties

Newton K., & Zmerli, S. (2011). Three forms of trust and their association. Euro Pol Sci Rev; 3: 169-200

1. **Trust in OutGroups**

The following statements were selected to assess trust outgroups. Participants were asked – “Could you tell us for each group listed below the extent to which you trust people from each group?”. These were scored on a 5 point scale where 1 = Do not trust at all, 7 Trust completely), and were taken from Newton and Zmerli (2011)

- People of another religion
- People of another nationality
- People you meet for the first time
- A stranger

Newton K., & Zmerli, S. (2011). Three forms of trust and their association. Euro Pol Sci Rev; 3: 169-200

1. **Trust in Individuals**

The following statements were selected to assess trust outgroups. Participants were asked – “Could you tell us for each group listed below the extent to which you trust people from each group?”. These were scored on a 4 point scale (where 1 = Strongly Disagree, 2 = Disagree, 3 Agree, 4 = Strongly Agree) , 7 Trust completely), and were taken from Dohmen, Falk, Huffman, Sunde (2008)

- In general, one can trust people
- When dealing with strangers, it is better to be careful before you trust them

Dohmen T, Falk A, Huffman D, Sunde U. (2008). Representative Trust and Reciprocity: Prevalence and Determinants. *Econ Inq*; 46: 84-90

# Supplementary File S5: Standardizing Trust Scores

Trust scores were standardised using the following linear transform:

$$normalize{d score}_{i}=\frac{score_{i}-minscore}{maxscore-minscore}$$

Thus, a score of 0 equates to no trust at all or complete lack of distrust, and 1 equates to complete trust or distrust. Scores in between these values indicate the degree of trust or distrust based on the re-scaled range (*max-min*). As this is a standard linear transform, it does not alter the relationship between predictors and outcomes; rather, it aids interpretation.

# Supplementary File S6: Standardized Trust Scores as a Function of Ethnicity and Donor Status

Table S2 below provides the mean, error, and 95% C.I.s for the six domains of trust as a function of ethnicity and donor status.

**Table S2**. Descriptive Statistics for the Dimensions of Trust by Donor Status and Ethnicity

| **Dimension of Trust** | **Current Donor** | **Ethnicity** | **Mean** | **Std. Error** | **95% Confidence Interval** | |
| --- | --- | --- | --- | --- | --- | --- |
|  |  |  |  |  | Lower | Upper |
| NHS and staff | No | Asian | .669 | .029 | .612 | .726 |
|  |  | Black | .645 | .023 | .599 | .691 |
|  |  | Mixed | .602 | .032 | .538 | .666 |
|  |  | White | .705 | .017 | .671 | .738 |
|  | Yes | Asian | .719 | .014 | .691 | .747 |
|  |  | Black | .684 | .019 | .647 | .721 |
|  |  | Mixed | .745 | .014 | .718 | .772 |
|  |  | White | .755 | .009 | .737 | .773 |
| NHSBT | no | Asian | .772 | .027 | .719 | .825 |
|  |  | Black | .715 | .022 | .672 | .758 |
|  |  | Mixed) | .648 | .030 | .589 | .708 |
|  |  | White | .790 | .016 | .759 | .821 |
|  | yes | Asian | .867 | .013 | .841 | .893 |
|  |  | Black | .853 | .018 | .819 | .888 |
|  |  | Mixed | .880 | .013 | .854 | .905 |
|  |  | White | .893 | .009 | .876 | .910 |
| Conditional  distrust | no | Asian | .340 | .028 | .284 | .395 |
|  |  | Black | .494 | .023 | .449 | .539 |
|  |  | Mixed | .469 | .032 | .406 | .531 |
|  |  | White | .269 | .017 | .236 | .301 |
|  | yes | Asian | .286 | .014 | .258 | .314 |
|  |  | Black | .444 | .019 | .408 | .481 |
|  |  | Mixed | .281 | .013 | .254 | .307 |
|  |  | White | .223 | .009 | .206 | .241 |
| Individuals | no | Asian | .366 | .032 | .302 | .429 |
|  |  | Black | .315 | .026 | .263 | .366 |
|  |  | Mixed | .399 | .036 | .328 | .471 |
|  |  | White | .500 | .019 | .463 | .537 |
|  | yes | Asian | .432 | .016 | .400 | .463 |
|  |  | Black | .372 | .021 | .331 | .414 |
|  |  | Mixed | .468 | .015 | .438 | .498 |
|  |  | White | .480 | .010 | .460 | .500 |
| Outgroup | no | Asian | .673 | .029 | .615 | .730 |
|  |  | Black | .603 | .024 | .557 | .650 |
|  |  | Mixed | .671 | .033 | .607 | .736 |
|  |  | White | .702 | .017 | .668 | .736 |
|  | yes | Asian | .686 | .015 | .657 | .714 |
|  |  | Black | .656 | .019 | .619 | .694 |
|  |  | Black | .656 | .019 | .619 | .694 |
|  |  | White | .705 | .009 | .687 | .723 |
| Politics | no | Asian | .368 | .042 | .285 | .451 |
|  |  | Black | .317 | .034 | .250 | .384 |
|  |  | Mixed | .333 | .047 | .240 | .427 |
|  |  | White | .365 | .025 | .316 | .414 |
|  | yes | Asian | .344 | .021 | .303 | .385 |
|  |  | Black | .278 | .028 | .224 | .332 |
|  |  | Mixed | .304 | .020 | .264 | .343 |
|  |  | White | .353 | .013 | .327 | .380 |

**Note**. Asian = People from Asian ethnic backgrounds, Black = People from Black and Caribbean backgrounds, Mixed = People from mixed ethnic backgrounds, White = People from White backgrounds

# Supplementary File S7: Anaysis of Margins and Sensitivity Analyses

**Margin for Regression in Table 3.** Table S3 below privudes the margins for the intercations reported in Table 3 in the main paper and supporting text.

**Table S3**: *Marginal Effects Analysis for Decomposition of the Interactions*

|  | | NHS & Staff | | | | NHSBT | | | Individuals | | |
| --- | --- | --- | --- | --- | --- | --- | --- | --- | --- | --- | --- |
|  | | dy/dx (se) | p | | 95% CI | dy/dx (se) | p | 95% CI | dy/dx (se) | p | 95% CI |
| Donor vs Non-Donor | |  |  | |  |  |  |  |  |  |  |
| Asian | | 0.056 (0.034) | .145 | | -0.019, 0.132 | **0.127 (0.031)** | **.001** | **0.058, 0.187** | 0.050 (0.040) | .211 | -0.028, 0.128 |
| Black | | 0.044 (0.035) | .218 | | -0.026, 0.115 | **0.151 (0.037)** | **.000** | **0.078, 0.225** | 0.065 (0.033) | .052 | -0.005, 0.131 |
| Mixed | | **0.125 (0.034)** | **.000** | | **0.058, 0.192** | **0.209 (0.041)** | **.000** | **0.128, 0.290** | **0.080 (0.038)** | **.036** | **0.005, 0.155** |
| White | | **0.043 (0.021)** | **.039** | | **0.002, 0.085** | **0.100 (0.020)** | **.000** | **0.061, 0.138** | -0.024 (0.021) | .257 | -0.067, 0.018 |
|  | Out-group | | | | | Political Process | | | Conditional Distrust | | |
|  | dy/dx (se) | | p | 95% CI | | dy/dx (se) | p | 95% CI | dy/dx (se) | p | 95% CI |
| Donor vs Non-Donor |  | |  |  | |  |  |  |  |  |  |
| Asian | 0.033 (0.036) | | .364 | -0.039, 0.105 | | -0.028 (0.060) | .636 | -0.143, 0.088 | -0.047 (0.036) | .197 | -0.118, 0.025 |
| Black | 0.059 (0.039) | | .118 | -0.015, 0.133 | | -0.004 (0.049) | .933 | -0.099, 0.091 | -0.069 (0.040) | .088 | -0.149, 0.010 |
| Mixed | 0.063 (0.032) | | .053 | -0.0007, 0.128 | | -0.047 (0.048) | .329 | -0.142, 0.047 | **-0.178 (0.040)** | **.000** | **-0.257, -0.099** |
| White | 0.013 (0.120) | | .510 | -0.026, 0.052 | | -0.004 (0.027) | .892 | -0.058, 0.051 | **-0.052 (0.018)** | **.004** | **-0.089, -0.017** |

**Note**. Asian = People from Asian ethnic backgrounds, Black = People from Black and Caribbean backgrounds, Mixed = People from mixed ethnic backgrounds, White = People from White backgrounds (excluding White minorities)

**Sensitivity Analysis**: The analyses in the tables below do not include the Windrush variable. This results in larger sample sizes and shows that while Windrush perceptions are important to trust, their inclusion does not alter the pattern of results.

**Table S4**. *Predictors of Trust and Distrust*

|  | Trust | | | | | | | | |
| --- | --- | --- | --- | --- | --- | --- | --- | --- | --- |
|  | NHS & Staff | | | NHSBT | | | Others | | |
|  | B | P= | 95% CI | B | P= | 95% CI | B | P= | 95% CI |
| Age | -0.0007 (0.0004) | .120 | -0.0015, 0.0001 | -0.0006 (0.0004) | .094 | -0.001, 0.0001 | **0.003 (0.0005)** | **.000** | **0.002, 0.004** |
| Sex | **0.036 (0.011)** | **.002** | **0.013, 0.059** | 0.003 (0.010) | .779 | -0.018, 0.023 | **0.045 (0.013)** | **.001** | **0.019, 0.070** |
| Current Donor | **0.042(0.020)** | **.033** | **0.003, 0.082** | **0.101 (0.019)** | **.000** | **0.063, 0.138** | -0.027 (0.020) | .181 | -0.068, 0.013 |
| Ethnicity |  |  |  |  |  |  |  |  |  |
| Asian | -0.054 (0.037) | .151 | -0.127, 0.012 | -0.042 (0.032) | .201 | -0.106, 0.022 | **-0.116 (0.038)** | **.002** | **-0.191, -0.041** |
| Black | **-0.077 (0.035)** | **.029** | **-0.146, -0.008** | **-0.096 (0.038)** | **.012** | **-0.171, -0.021** | **-0.153 (0.033)** | **.000** | **-0.219, -0.088** |
| Mixed | **-0.112 (0.036)** | **.002** | **-0.084, -0.041** | **-0.147 (0.042)** | **.000** | **-0.230, -0.064** | **-0.078 (0.039)** | **.046** | **-0.155, -0.001** |
| Interaction |  |  |  |  |  |  |  |  |  |
| Donor+Asian | 0.002 (0.040) | .949 | -0.076, 0.080 | 0.003(0.036) | .932 | -0.067, 0.073 | **0.093 (0.041)** | **.025** | **0.012, 0.175** |
| Donor+Black | 0.003 (0.040) | .946 | -0.077, 0.082 | 0.050 (0.041) | .222 | -0.031, 0.131 | **0.082 (0.039)** | **.037** | **0.005, 0.160** |
| Donor+Mixed | **0.089 (0.039)** | **.022** | **0.013, 0.166** | **0.117 (0.044)** | **.008** | **0.030, 0.204** | **0.112 (0.042)** | **.009** | **0.029, 0.195** |
| BREXIT | **-0.041 (0.013)** | **.002** | **-0.067, -0.016** | **-0.034 (0.012)** | **.006** | **-0.059, -0.010** | **-0.030 (0.013)** | **.023** | **0.005, 0.154** |
| Constant | **0.749 (0.028)** | **.000** | **0.696, 0.804** | **0.839 (0.027)** | **.000** | **0.786, 0.893** | **0.334 (0.030)** | **.000** | **0.275, 0.394** |
| R2 | .07 |  |  | .147 |  |  | .181 |  |  |
| N | 928 |  |  | 931 |  |  | 922 |  |  |

|  | Trust | | | | | | Distrust | | |
| --- | --- | --- | --- | --- | --- | --- | --- | --- | --- |
|  | Out-Group | | | Political Process | | | Conditional Distrust | | |
|  | B | P= | 95% CI | B | P= | 95% CI | B | P= | 95% CI |
| Age | **0.001 (0.0004)** | **.014** | **0.0002, 0.009** | 0.0004 (0.0006) | .538 | -0.0009,0.002 | **-0.0008 0.0004)** | **.045** | **-0.002, -0.0003** |
| Sex | -0.001 (0.012) | .901 | -0.025, 0.022 | 0.026(0.018) | .886 | -0.032, 0.038 | -0.038 (0.012) | .744 | -0.027, 0.019 |
| Current Donor | 0.008 (0.019) | .668 | -0.029, 0.045 | -0.008 (0.027) | .777 | -0.061, 0.045 | **-0.048 (0.017)** | **.007** | **-0.082, -0.013** |
| Ethnicity |  |  |  |  |  |  |  |  |  |
| Asian | -0.025 (0.034) | ..464 | -0.094, 0.042 | 0.031 (0.060) | .605 | -0.087, 0.149 | 0.051 (0.036) | .164 | -0.020, 0.122 |
| Black | **-0.094 (0.035)** | **.008** | **-0.164, -0.024** | -0.039 (0.048) | .418 | -0.134, -0.056 | **0.222( 0.039)** | **.000** | **0.146, 0.298** |
| Mixed | -0.023 (0.034) | .493 | -0.089, 0.043 | -0.007(0.052) | .887 | -0.109, 0.095 | **0.180 (0.043)** | **.000** | **0.095, 0.266** |
| Interaction |  |  |  |  |  |  |  |  |  |
| Donor+Asian | 0.009 (0.038) | .815 | -0.076, 0.083 | -0.025(0.064) | .696 | -0.150, 0.100 | 0.014 (0.039) | .711 | -0.062, 0.091 |
| Donor+Black | 0.046 (0.042) | .274 | -0.036, 0.129 | -0.007 (0.057) | .892 | -0.121, 0.105 | -0.022 (0.045) | .624 | -0.109, 0.066 |
| Donor+Mixed | 0.062 (0.037) | .091 | -0.010, 0.134 | -0.022 (0.056) | .692 | -0.132, 0.087 | **-0.134 (0.046)** | **.003** | **-0.223, -0.044** |
|  |  |  |  |  |  |  |  |  |  |
| BREXIT | **-0.041 (0.013)** | **.002** | **-0.066, -0.016** | 0.027 (0.019) | .159 | -0.010, 0.064 | **-0.030 (0.013)** | **.023** | **0.005, 0.156** |
| Constant | **0.658 (0.028)** | **.000** | **0.602, 0.713** | **0.329 (0.041)** | **.000** | **0.249, 0.410** | **0.032 (0.012)** | **.009** | **0.110, 0.350** |
| R2 | .170 |  |  | .012 |  |  | .163 |  |  |
| N | 933 |  |  | 931 |  |  | 925 |  |  |

**Note**. Sex (0 = female, 1 = male). BAME Status: People from a White background are the comparison population. BREXIT (0 = negative influence, 1 = positive influence). Asian = People from Asian ethnic backgrounds, Black = People Black and Caribbean backgrounds, Mixed = People from mixed ethnic backgrounds, White = People from White backgrounds (excluding White minorities).

**Table S5**: *Marginal Effects Analysis for Decomposition of the Interactions*

|  | NHS & Staff | | | NHSBT | | | Others | | |
| --- | --- | --- | --- | --- | --- | --- | --- | --- | --- |
|  | dy/dx (se) | p | 95% CI | dy/dx (se) | p | 95% CI | dy/dx (se) | p | 95% CI |
| Donor vs Non-Donor |  |  |  |  |  |  |  |  |  |
| Asian | 0.045 (0.034) | .188 | -0.22, 0.113 | **0.103 (0.030)** | **.001** | **0.045, 0.162** | 0.066 (0.036) | .071 | -0.005, 0.137 |
| Black | 0.046 (0.035) | .202 | -0.24, 0.115 | **0.151 (0.037)** | **.000** | **0.079, 0.223** | 0.055 (0.034) | .109 | -0.012, 0.122 |
| Mixed | **0.132 (0.033)** | **.000** | **0.066, 0.198** | **0.217 (0.040)** | **.000** | **0.138, 0.296** | **0.084 (0.037)** | **.024** | **0.011, 0.158** |
| White | **0.043 (0.020)** | **.033** | **0.003, 0.082** | **0.100 (0.018)** | **.000** | **0.063, 0.137** | -0.027 (0.020) | .181 | -0.068, 0.013 |
|  | Out-group | | | Political Process | | | Conditional Distrust | | |
|  | dy/dx (se) | p | 95% CI | dy/dx (se) | p | 95% CI | dy/dx (se) | p | 95% CI |
| Donor vs Non-Donor |  |  |  |  |  |  |  |  |  |
| Asian | 0.017 (0.033) | .607 | -0.048, 0.082 | -0.032 (0.058) | .574 | -0.147, 0.081 | -0.033 (0.035) | .343 | -0.102, 0.035 |
| Black | 0.054 (0.038) | .153 | -0.020, 0.128 | -0.015 (0.051) | .762 | -0.116, 0.085 | -0.069 (0.041) | .093 | -0.151, 0.012 |
| Mixed | **0.070 (0.032)** | **.026** | **0.008, 0.132** | -0.030 (0.049) | .544 | -0.126, 0.067 | **-0.181 (0.042)** | **.000** | **-0.264, -0.099** |
| White | 0.008 (0.018) | .668 | -0.029, 0.045 | -0.007 (0.027) | .777 | -0061, 0.045 | **-0.048 (0.017)** | **.007** | **-0.082, -0.013** |

**Note**. Asian = People from Asian ethnic backgrounds, Black = People from Black and Caribbean backgrounds, Mixed = People from mixed ethnic backgrounds, White = People from White backgrounds (excluding White minorities).

# Supplementary File S8: Analysis of the Cultural Context of the Study: BREXIT & Windrush Scandal

**Cultural Context**

In terms of the cultural context seventy-four (55% White people, 25% people from an Asian background, 17% people from a mixed ethnic background & 3% people from a Black and Caribbean background) people stated that they were not sure what the Windrush Scandal was. Of those who had awareness, the mean was 3.79 (SD = 1.03) and of those who agreed more strongly that the Windrush Scandal were more likely to endorse ‘condition distrust’ (r = .311, p = .000, n = 893) and be less trusting of ‘others’ (r = -.108, p = .001, n = 890) and ‘politics’ (r = -.216, p = .000, n = 897).

Two-hundred-and-sixty-eight (23.7%) thought that Brexit is a positive step for the future of the UK and 699 (72.3%) that is a negative step. Those who perceive Brexit as a positive step are less trusting of ‘NHS and its staff’ (ρ = -.087, p = .007, n = 958), ‘NHSBT’ (ρ = -.085, p = .009, n = 961), ‘outgroups’ (ρ = -.073, p = .024, n = 863 and more trusting of ‘politics’ (ρ = .064, p = .049, n = 961). Thus, perceiving Brexit as a positive thing for the UK is associated with a lack of trust in UK established institutions but greater trust in politics. The trust in politics may represent support for the government negotiations for BREXIT at the time of the survey. The lack of trust in politics for those who perceive the Windrush Scandal as an endorsement that the UK government has a negative view of minorities may represent alienation from the political process.

# Supplementary File S9: Details of Path Models For the Indirect Effect of Perceptions of the Brexit and the Windrush Scandal on the demographic-trust link

**The Model**. The conceptual model for the path analysis is shown in Figure S1. In this model, age, sex (0 = female, 1 = male) and ethnicity (0 = White people, 1 = all ethnic minorities), were specified to predict perceptions of Brexit and the Windrush Scandal, the six trust dimensions and perceptions of Brexit and the Windrush Scandal were also specified to predict the six trust dimensions. Perceptions of Brexit and the Windrush Scandal were specified as correlated.
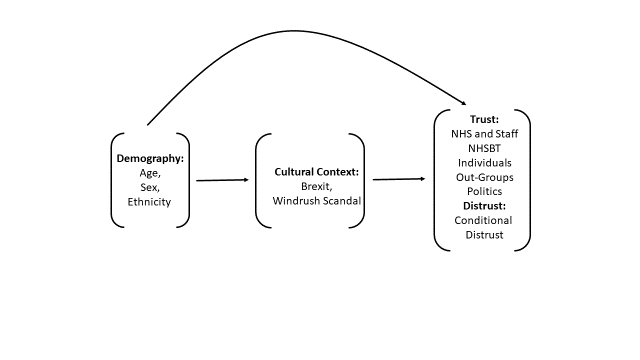


**Figure S1**: *Conceptual Mediation Model*

**Model Fit**. This model had an excellent fit to these data (CFI = 1.0, TLI = 0.997, RMSAE = 0.010 (p RMSEA <= .05 = .808).

**Predicting Brexit and the Windrush Scandal.** The path coefficients are presented in Table S5. As shown, older participants had a greater belief that Brexit was a positive change for the UK’, and people from ethnic minorities and women perceived that the Windrush scandal indicated that the UK government still held negative views about ethnic minorities.

**Predicting Trust**. The predictors of trust are as they are in Table 3 and 4 in the main text. Trust in NHS and Staff is greater for those who see Brexit as a negative change for the UK, are male, white, and current blood donors. Trust in NHSBT is greater for those who see Brexit as a negative change for the UK, White people, and current blood donors. Conditional distrust is greater for those who see Brexit as a positive change, and the Windrush scandal indicates that the UK Government has a negative view of ethnic minorities still, people from all ethnic minorities and non-donors. Trust in individuals was greater for those who see Brexit as a negative change for the UK, those who see the Windrush scandal as less likely to indicate that the UK government has a negative view of ethnic minorities, being older, male, White and a current blood donor. Trust in out-groups is greater for those who see Brexit as a negative change for the UK, White people, and current blood donors. Trust in politics is greater for those less likely to see the Windrush scandal as indicating that the UK government has a negative view of ethnic minorities.

**Indirect Effects of Perception of Brexit and the Windrush Scandal**. Table S6 details perceptions of Brexit and the Windrush Scandal as indirectly linking demographics to trust. The analyses targeted demographics that had associations with perceptions of Brexit and/or the Windrush Scandal, as these were the variables that perceptions of Brexit and the Windrush Scandal could act on as indirect mechanisms.

Older people were more likely to indicate that Brexit was beneficial to the UK, and this greater sense of benefit was linked to lower trust in the NHS and Staff, NHSBT, individuals, and out-groups, as well as greater conditional distrust.

People from ethnic minority communities displayed greater conditional distrust and reduced trust in individuals and politics because of greater perceptions that the Windrush Scandal indicated that the UK government still held negative views of people from ethnic minority backgrounds.

Women have greater condition distrust because women are more likely to perceive that the Windrush Scandal indicates that the UK government still held negative views of people from ethnic minority backgrounds. Women also have less trust in politics via the same mechanism relating to perceptions of the Windrush Scandal.

Thus, perceptions that Brexit is beneficial for the UK is a particularly important mechanism that links increased age to reduced trust. Whereas perceptions that the Windrush Scandal indicated that the UK government still holds negative views of people from ethnic minority backgrounds are critical, linking being a woman and being from an ethnic minority community to reduced trust and increased conditional distrust.

**Table S6**: *Path Model Estimates for Mediation Model*

|  | Estimate | S.E. | Est./S.E. | P-Value |
| --- | --- | --- | --- | --- |
| **BREXIT** |  |  |  |  |
| Ethnicity | -0.046 | 0.052 | -0.879 | 0.380 |
| Age | 0.336 | 0.050 | 6.679 | 0.000 |
| Sex | 0.065 | 0.045 | 1.452 | 0.147 |
| **Windrush Scandal** |  |  |  |  |
| Ethnicity | 0.328 | 0.034 | 9.770 | 0.000 |
| Age | -0.071 | 0.037 | -1.944 | 0.052 |
| Sex | -0.099 | 0.032 | -3.088 | 0.002 |
| **Trust in NHS and its Staff** |  |  |  |  |
| BREXIT | -0.126 | 0.053 | -2.376 | 0.018 |
| Windrush Scandal | -0.037 | 0.037 | -0.983 | 0.326 |
| Age | -0.054 | 0.044 | -1.235 | 0.217 |
| Sex | 0.116 | 0.036 | 3.241 | 0.001 |
| Ethnicity | -0.166 | 0.040 | -4.114 | 0.000 |
| Current Blood Donor | 0.146 | 0.032 | 4.520 | 0.000 |
| **Trust in NHSBT** |  |  |  |  |
| BREXIT | -0.128 | 0.052 | -2.461 | 0.014 |
| Windrush Scandal | -0.051 | 0.039 | -1.324 | 0.185 |
| Age | -0.079 | 0.043 | -1.821 | 0.069 |
| Sex | 0.036 | 0.036 | 0.978 | 0.328 |
| Ethnicity | -0.160 | 0.040 | -3.977 | 0.000 |
| Current Blood Donor | 0.317 | 0.029 | 10.899 | 0.000 |
| **Conditional Distrust** |  |  |  |  |
| BREXIT | 0.188 | 0.051 | 3.678 | 0.000 |
| Windrush Scandal | 0.278 | 0.037 | 7.545 | 0.000 |
| Age | -0.077 | 0.040 | -1.913 | 0.056 |
| Sex | -0.019 | 0.034 | -0.555 | 0.579 |
| Ethnicity | 0.220 | 0.040 | 5.440 | 0.000 |
| Current Blood Donor | -0.212 | 0.030 | -7.146 | 0.000 |
| **Trust in Individuals** |  |  |  |  |
| BREXIT | -0.163 | 0.055 | -2.969 | 0.003 |
| Windrush Scandal | -0.093 | 0.040 | -2.321 | 0.020 |
| Age | 0.244 | 0.041 | 5.916 | 0.000 |
| Sex | 0.115 | 0.034 | 3.339 | 0.001 |
| Ethnicity | -0.082 | 0.039 | -2.081 | 0.037 |
| Current Blood Donor | 0.074 | 0.033 | 2.229 | 0.026 |
| **Trust on Out-Groups** |  |  |  |  |
| BREXIT | -0.132 | 0.055 | -2.397 | 0.017 |
| Windrush Scandal | -0.010 | 0.041 | -0.233 | 0.815 |
| Age | 0.043 | 0.043 | 1.003 | 0.316 |
| Sex | -0.008 | 0.038 | -0.224 | 0.823 |
| Ethnicity | -0.112 | 0.041 | -2.750 | 0.006 |
| Current Blood Donor | 0.106 | 0.033 | 3.241 | 0.001 |
| **Trust in Politics** |  |  |  |  |
| BREXIT | -0.018 | 0.055 | -0.331 | 0.741 |
| Windrush Scandal | -0.207 | 0.039 | -5.330 | 0.000 |
| Age | 0.042 | 0.042 | 0.999 | 0.318 |
| Sex | -0.004 | 0.035 | -0.101 | 0.920 |
| Ethnicity | -0.005 | 0.040 | -0.134 | 0.894 |
| Current Blood Donor | -0.027 | 0.034 | -0.792 | 0.429 |

**Note**. *Brexit* (Do you think the Brexit is a positive or negative step for the future of the UK? (0 = negative, 1 = positive); Ethnicity (0 = People from White backgrounds, 1 = People from all ethnic minorities); *Windrush Scandal* (The Windrush scandal shows that the authorities still have a negative view about ethnic minorities in the UK? 1= strongly disagree, to 5 Strongly agree); *Sex* (0 = female, 1 = male); *Current Blood Donor* (0 = not current donor, 1 = current donor)

**Table S7:** *Indirect effect of age, sex and ethnicity via Windrush Scandal and Brexit on estimates of Trust*

| Trust in NHS & Staff | | | | |
| --- | --- | --- | --- | --- |
|  | Trust in NHS & Staff form Ethnicity via Windrush Scandal. | | | |
|  | Estimate | S.E. | Estimate/S.E. | P-value |
| **Ethnicity via Windrush Scandal** | -0.004 | 0.004 | -0.973 | 0.331 |
|  | -.002 (standardised) |  |  |  |
|  | Trust in NHS & Staff from Sex via Windrush Scandal. | | | |
|  | Estimate | S.E. | Estimate/S.E. | P-value |
| **Sex via Windrush Scandal** | 0.001 | 0.001 | 0.940 | 0.347 |
|  | .004 (standardized) |  |  |  |
|  | Trust in NHS & Staff form age via Brexit. | | | |
|  | Estimate | S.E. | Estimate/S.E. | P-value |
| **Age via Brexit** | **-0.001** | **0.000** | **-2.219** | **0.027** |
|  | -.042 (standardised) |  |  |  |
|  | Trust in NHS & Staff form age via Windrush Scandal | | | |
|  | Estimate | S.E. | Estimate/S.E. | P-value |
| **Age via Windrush Scandal** | 0.000 | 0.000 | 0.874 | 0.382 |
|  | .003 (standardized) |  |  |  |
|  |  |  |  |  |
| Trust in NHSBT | | | | |
|  | Trust in NHSBT form ethnicity via Windrush Scandal | | | |
|  | Estimate | S.E. | Estimate/S.E. | P-value |
| **Ethnicity via Windrush Scandal** | -0.006 | 0.004 | -1.307 | 0.191 |
|  | -.017 (standardised) |  |  |  |
|  | Trust in NHSBT form sex via Windrush Scandal | | | |
|  | Estimate | S.E. | Estimate/S.E. | P-value |
| **Sex via Windrush Scandal** | 0.002 | 0.001 | 1.216 | 0.224 |
|  | .005 (standardized) |  |  |  |
|  | Trust in NHSBT form age via Brexit | | | |
|  | Estimate | S.E. | Estimate/S.E. | P-value |
| **Age via Brexit** | **-0.001** | **0.000** | **-2.299** | **0.021** |
|  | -.041 (standardised) |  |  |  |
|  | Trust in NHSBT form age via Windrush Scandal | | | |
|  | Estimate | S.E. | Estimate/S.E. | P-value |
| **Age via Windrush Scandal** | 0.000 | 0.000 | 1.083 | 0.279 |
|  | .004 (standardized) |  |  |  |
|  |  |  |  |  |
| Conditional Distrust | | | | |
|  | Conditional Distrust form ethnicity via Windrush Scandal | | | |
|  | Estimate | S.E. | Estimate/S.E. | P-value |
| **Ethnicity via Windrush Scandal** | **0.034** | **0.006** | **5.517** | **0.000** |
|  | .091 (standardized) |  |  |  |
|  | Conditional Distrust form sex via Windrush Scandal | | | |
|  | Estimate | S.E. | Estimate/S.E. | P-value |
| **Sex via Windrush Scandal** | **-0.011** | **0.004** | **-2.799** | **0.005** |
|  | -.028 (standardised) |  |  |  |
|  | Conditional Distrust form age via Brexit | | | |
|  | Estimate | S.E. | Estimate/S.E. | P-value |
| **Age via Brexit** | **0.001** | **0.000** | **3.188** | **0.001** |
|  | .063 (standardized) |  |  |  |
|  | Conditional Distrust form age via Windrush Scandal | | | |
|  | Estimate | S.E. | Estimate/S.E. | P-value |
| **Age via Windrush Scandal** | -0.000 | 0.000 | -1.876 | 0.061 |
|  | -.020 (standardised) |  |  |  |
|  |  |  |  |  |
| Trust in Individuals | | | | |
|  | Trust in Individuals from ethnicity via Windrush Scandal | | | |
|  | Estimate | S.E. | Estimate/S.E. | P-value |
| **Ethnicity via Windrush Scandal** | **-0.012** | **0.005** | **-2.228** | **0.026** |
|  | -.030 (standardised) |  |  |  |
|  | Trust in Individuals from sex via Windrush Scandal. | | | |
|  | Estimate | S.E. | Estimate/S.E. | P-value |
| **Sex via Windrush Scandal** | 0.004 | 0.002 | 1.810 | 0.070 |
|  | .009 (standardized) |  |  |  |
|  | Trust in Individuals from age via Brexit | | | |
|  | Estimate | S.E. | Estimate/S.E. | P-value |
| **Age via Brexit** | **-0.001** | **0.000** | **-2.668** | **0.008** |
|  | -.055(standardised) |  |  |  |
|  | Trust in Individuals from age via Windrush Scandal | | | |
|  | Estimate | S.E. | Estimate/S.E. | P-value |
| **Age via Windrush Scandal** | 0.000 | 0.000 | 1.477 | 0.140 |
|  | .007 (standardized) |  |  |  |
|  |  |  |  |  |
| Trust in Out-Groups | | | | |
|  | Trust in Out-Groups from ethnicity via Windrush Scandal | | | |
|  | Estimate | S.E. | Estimate/S.E. | P-value |
| **Ethnicity via Windrush Scandal** | -0.001 | 0.005 | -0.233 | 0.816 |
|  | -.003 (standardised) |  |  |  |
|  | Trust in Out-Groups from sex via Windrush Scandal | | | |
|  | Estimate | S.E. | Estimate/S.E. | P-value |
| **Sex via Windrush Scandal** | 0.000 | 0.001 | 0.232 | 0.816 |
|  | .001 (standardized) |  |  |  |
|  | Trust in Out-Groups from age via Brexit | | | |
|  | Estimate | S.E. | Estimate/S.E. | P-value |
| **Age via Brexit** | **-0.001** | **0.000** | **-2.246** | **0.025** |
|  | -.044 (standardised) |  |  |  |
|  | Trust in Out-Groups from age via Windrush Scandal | | | |
|  | Estimate | S.E. | Estimate/S.E. | P-value |
| **Age via Windrush Scandal** | 0.000 | 0.000 | 0.232 | 0.817 |
|  | .001 (standardized) |  |  |  |
|  |  |  |  |  |
| Trust in Politics | | | | |
|  | Trust in Politics from ethnicity via Windrush Scandal | | | |
|  | Estimate | S.E. | Estimate/S.E. | P-value |
| **Ethnicity via Windrush Scandal** | **-0.034** | **0.008** | **-4.429** | **0.000** |
|  | -.068 (standardised) |  |  |  |
|  | Trust in Politics from sex via Windrush Scandal | | | |
|  | Estimate | S.E. | Estimate/S.E. | P-value |
| **Sex via Windrush Scandal** | **0.011** | **0.004** | **2.646** | **0.008** |
|  | .020 (standardized) |  |  |  |
|  | Trust in Politics from age via Brexit | | | |
|  | Estimate | S.E. | Estimate/S.E. | P-value |
| **Age via Brexit** | -0.000 | 0.000 | -0.331 | 0.741 |
|  | -.006 (standardised) |  |  |  |
|  | Trust in Politics from age via Windrush Scandal | | | |
|  | Estimate | S.E. | Estimate/S.E. | P-value |
| **Age via Windrush Scandal** | 0.000 | 0.000 | 1.792 | 0.073 |
|  | .015 (standardized) |  |  |  |

# Supplementary File S10: Examination of the interaction of ethnicity by trust to predict willingness to donate in non-donors

In the main analyses, Table 4 shows two significant moderating effects of ethnicity on trust, one for “Trust in NHSBT” and one for “Trust in Individuals”. .

Tables S8 and S9 show the margins analyses for the two significant interactions for Table 4 for columns 2 and 3. The first Table (Table S8) shows the margins for the interaction of ethnicity with ‘trust in individuals’, and the second (Table S9) shows the margins for the interaction of ethnicity with ‘trust in NHSBT’,. These analyses show that for people from ethnic minority backgrounds, greater trust in individuals predicts willingness to donate and that for White people, greater ‘trust in NHSBT’ predicts willingness to donate.

**Table S8:** *Margins for the ethnicity by ‘trust in individuals’ interaction on willingness to donate in non-donors*

|  | Delta-method | | | 95% C.I. | |
| --- | --- | --- | --- | --- | --- |
|  | dy/dx | std. err. | p = | Lower | Upper |
|  |  |  |  |  |  |
| Trust in Individual |  |  |  |  |  |
| Ethnicity |  |  |  |  |  |
| People from White backgrounds | -0.1000722 | 0.2237121 | .655 | -0.5385398 | 0.3383955 |
| **People from all ethnic minority backgrounds** | **0.584251** | **0.1779937** | **.001** | **0.2353898** | **0.9331122** |

**Table S9:** *Margins for the ethnicity by ‘trust in NHSBT’interaction on willingness to donate in non-donors*

|  | Delta-method | | | 95% C.I. | |
| --- | --- | --- | --- | --- | --- |
|  | dy/dx | std. err. | p = | Lower | Upper |
|  |  |  |  |  |  |
| Trust in NHSBT |  |  |  |  |  |
| Ethnicity |  |  |  |  |  |
| **People from White backgrounds** | **0.5456031** | **0.2536743** | **.031** | **0.0484106** | **1.042796** |
| People from all ethnic minority backgrounds | -0.1429955 | 0.2356642 | .544 | -0.6048889 | 0.3188978 |

# Supplementary File S11: Detail of Summary Path Models For the Prediction of Willingness to Donate in Non-Donors

**The Model:** While there were no direct effects of perceptions of Brexit and the Windrush Scandal on willingness to donate in existing donors (see Supplementary File S2, and main tables 3 and 4), we explored if they have indirect effects on willingness to donate in non-donors via the two dimensions of trust identified in Table 5 in the main text as predictors of willingness: trust in individuals and NHSBT.

To account for the interaction of these trust dimensions with ethnicity, the model was separately specified for White people and ethnic minority communities. Perceptions of Brexit and the Windrush Scandal were specified as correlated, as were trust in NHSBT and individuals.

The conceptual model is shown in Figure S2.


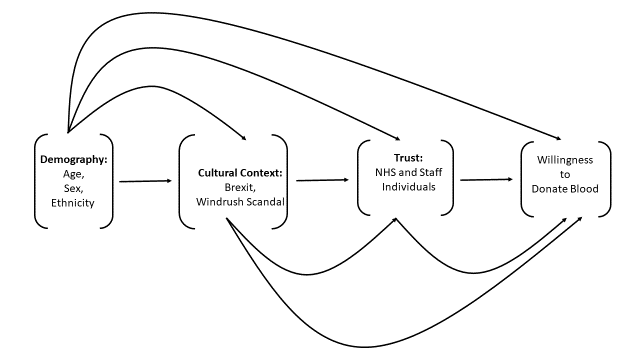


**Figure S2:** *Conceptual Model Predicting Willingness to Donate in Non-Donors*

**Model Fit:** This is a saturated model, so both the TLI and CFI = 1 and the RMSEA = 0.
